# Emergency preparedness for infant and young child feeding in emergencies (IYCF-E): an Australian audit of emergency plans and guidance

**DOI:** 10.1186/s12889-019-7528-0

**Published:** 2019-10-15

**Authors:** Karleen Gribble, Mary Peterson, Decalie Brown

**Affiliations:** 1 School of Nursing and Midwifery, Western Sydney University, Locked Bag 1797, Penrith, NSW 2751 Australia; 2 Australian Breastfeeding Association, Melbourne, Australia; 3 NSW Health, Sydney, Australia; 4 World Breastfeeding Trends Initiative Australia, https://wbtiaus.com/

**Keywords:** Emergencies, Disasters, Breastfeeding, Formula feeding, Artificial feeding, Infants, Children, Planning

## Abstract

**Background:**

Australia experiences a high incidence of natural emergencies and Australian governments have committed significant investment into emergency preparedness and response. Amongst the population groups most vulnerable to emergencies are infants and young children with their vulnerability centering around their specific food and fluid needs. For this reason, the World Health Assembly has urged all member states to develop and implement infant and young child feeding in emergency (IYCF-E) plans in line with international guidance. This study aimed to determine the degree to which Australia has complied with this direction by conducting an audit of Australian emergency plans and guidance.

**Methods:**

Australian Federal, State/Territory and a sample of Local government emergency plans and guidance were located via web searches. Documents were searched for key words to identify content dealing with the needs of infants and young children. Plans and guidance were also searched for content dealing with the needs of animals as a comparison.

**Results:**

While plans and guidance contained numerous pointers to the desirability of having plans that address IYCF-E, there was a dearth of planning at all levels of government for the needs of infants and young children. Guidance related to heat waves contained information that could prove dangerous to infants. No agency at Federal or State/Territory had designated responsibility for IYCF-E or children in general. This was in stark contrast to the situation of animals for which there was widespread and comprehensive planning at all levels of government with clear designation of organisational responsibility.

**Conclusions:**

Lack of planning for IYCF-E in Australia places infants and young children at serious risk of adverse health consequences in emergencies. Australian Federal, State/Territory and Local governments need to take action to ensure that IYCF-E plans and guidance are developed and deployed in line with international standards. The pathway to successful integration of animal welfare plans provides a method for a similar integration of IYCF-E plans. Government health authorities are best placed to lead and be responsible for IYCF-E in Australia. National governments internationally should similarly take action to ensure that their youngest, most vulnerable citizens are protected in emergencies.

## Background

Australia commonly experiences natural emergencies that threaten the lives of individuals and cause significant environmental and property damage as well as economic loss [1, 2]. Most natural emergencies in Australia are of a climatic nature; flood, bushfire, heatwave, and cyclone, and are likely to increase in prevalence and severity as a result of climate change [3]. For these reasons, Australian Federal, State/Territory as well as Local governments have invested significant resources into emergency preparedness [4, 5]. Emergency plans are an integral part of this emergency preparedness.

Emergencies do not impact all segments of the population equally; some groups are more vulnerable to disasters than others [6]. It is recognised that emergency plans should account for the special needs of vulnerable groups in order to ensure that the risk they face be appropriately mitigated and that appropriate assistance be provided [7]. Amongst the groups that are more vulnerable are women, those who experience disability, the elderly, the socioeconomically disadvantaged, and children [8–11]. For children, the younger the child, the more vulnerable they are with the youngest infants being the most vulnerable [12, 13].

Infants have very specific food and fluid requirements, an immature immune system, are vulnerable to dehydration and are dependent on others for their care needs [14]. These characteristics interact with environmental conditions associated with emergencies such as poor sanitation, food and water shortages, power shortages, overcrowding, and restricted access to health care, to create the situation where infants are at heightened risk [15].

Large-scale emergencies in developed and developing countries have seen infants and young children experience increased morbidity, and often mortality, in emergencies [16–19]. Such adverse outcomes are usually a result of gastrointestinal or respiratory tract infections and associated with malnutrition or dehydration. As was observed during the 2011 Brisbane flooding and Cyclone Yasi emergencies, Australia is not immune to these risks. Increased rates of illness, including gastrointestinal infections, as well as hospitalisations connected to infant feeding problems, were recorded in these emergencies [20]. The hot weather commonly associated with Australian emergencies, in particular bushfire and cyclone, increases the risks posed to the very young.

The danger at which infants are placed in an emergency is impacted by whether the child is breastfed or dependent on infant formula. In an emergency, infants and young children who are breastfed are in a position of relative strength. Provided mothers do not become dehydrated, their ability to produce milk will be unaffected by emergencies as neither lack of food (in the short term) nor stress, impact milk production [21]. This means that breastfeeding children have access to a safe supply of food and water. It also means that children are provided with the anti-infective and growth factors in breastmilk that provide external immune support, assist the development of the immune system, help to prevent infection, and aid recovery from infection [22].

In contrast, infants who are dependent on infant formula are in a vulnerable position. Resources necessary for feeding such as infant formula, clean water, electricity or gas for heating water, hygienic food preparation and washing environments, and health care may be difficult or impossible to access in an emergency [23, 24]. Furthermore, formula fed infants lack the external immune support provided by breastmilk, while aspects of the immune system of young infants can be compromised by the feeding of infant formula [22].

The caregivers of both breastfed and formula fed infants require targeted support in emergencies. The mothers of breastfed infants need assistance to continue breastfeeding despite the stress and other challenges associated with emergencies. The caregivers of formula fed infants require assistance to access the resources needed to formula feed with acceptable safety. Without such support, infants may be at serious risk. This was evident in Christchurch, New Zealand after the 2011 earthquake where it was identified that some caregivers were using unboiled mains water to reconstitute infant formula despite it being contaminated with sewage [25]. This was perhaps because power supplies were cut and caregivers did not have access to resources for heating water. Furthermore, a lack of appropriate control of donations allowed distribution of infant formula that was out-of-date and containing non-approved ingredients [26].

The imperative for infants and young children to receive targeted aid in emergencies resulted in the development of the international guidance document the Operational Guidance on Infant and Young Child Feeding in Emergencies (OG- IFE). The OG-IFE provides governments, aid organisations and individuals with direction on how to ensure that infants and young children are provided with the support they need in emergencies [27]. The OG-IFE requires that policies on infant and young child feeding be developed, that emergency relief and management staff be trained on these policies, and that interventions be planned and implemented to support breastfed, formula fed and complementary fed children [27].

In 2010 the World Health Assembly urged member states, including Australia, to ensure that they had national emergency preparedness plans in line with the OG-IFE [28]. In addition, in 2013 the Australian national infant feeding guidance, the Infant Feeding Guidelines for Health Workers, stated that emergency preparedness plans should be made regarding IYCF-E [29]. However, the extent to which Australian governments have planned for the needs of infants and young children in emergencies has not previously been considered.

Audits of IYCF-E planning have been carried out in other jurisdictions including in the UK where planning provision for infants was compared to that for animals. It was noted that, *“There is government guidance for providing for animals, including pets, livestock, and zoo and circus animals, but there is no mention of mothers and infants”* [30]. Australian recognition that animals require specific and detailed emergency planning is relatively new [31] however, in contrast to infants and young children, the extent of emergency planning for animals has previously been considered. An audit by Taylor et al. [32] found that while there were shortcomings, Australian national and state/territory jurisdictions had extensive legislation, plans, guidelines, and community engagement materials dealing with animals in emergencies.

This study conducted an audit of Australian emergency preparedness and response plans with the goal of determining the degree to which Australia has planned for the needs of infants and young children in emergencies. The extent to which animals were planned for was also examined in order to allow a direct comparison to a recently institutionalised aspect of emergency response. It was considered that this comparison might assist in identifying pathways for integration of planning for infants and young children should the audit reveal inadequacies. As Australia has three levels of government, Federal (Commonwealth), State/Territory, and Local, plans and guidance of all levels of government were considered.

## Methods

Australian emergency management plans and related policies and guidelines were audited for content related to infants and young children between March and October of 2018. National and State/Territory documents were initially identified by using the web search terms of “Australia”, “NSW”, “Victoria”, “Queensland”, “Western Australia”, “South Australia”, “Tasmania”, “ACT” “Northern Territory” AND “emergency management” to locate the relevant main government emergency management website. The Federal, State/Territory over-arching emergency management plan was then located by browsing the website. Any other emergency management plans or guidance identified while searching for the over-arching plan were also noted. The following terms were searched in plans and guidance: “child”, “infant”, “bab*”, “milk”, “breast”, “formula”, “food”, “catering”, “water”, “animal” and “pet”. As each plan was searched, references to related plans and guidance were noted and also searched. As these further plans were searched, references to other plans and guidance were noted and searched. This process continued until no additional documents were identified. Where plans were not publicly available, the relevant government organisation responsible for the plans was contacted and access requested. Documents that were identified as obviously not relevant to infants and young children (e.g. space re-entry debris plan or marine chemical spill contingency plan) were not searched.

When terms were located, the relevant text was copied and pasted into a Google Doc®. Excerpts containing the search term “water” were only included in relation to drinking water, contaminated water, or water for washing and food preparation and excerpts concerning animal disease were excluded. Notes were made on the relevance of the excerpted text to the needs of infant and young children as texts were inserted into the Google Doc®.

In relation to Local government plans, the Australian Emergency Management Disaster Hub database of disaster events was used to identify Local government areas that had experienced emergencies (excluding building fires, shipwrecks, small-scale infection outbreaks, and droughts). Local government areas that had experienced an emergency in the years 2010–2014 (the last year for which events were added to the database) were identified and searched using the same search terms. Emergency kit lists were located by searching the internet using the name of the State or Territory and the phrase “emergency kit.”

When all documents had been searched, notes made on the relevance of excerpts were used to identify text that had relevance to IYCF-E and these excerpts were analysed for content and meaning.

## Results

### National plans and guidance

Twenty-two current national emergency plans and guidance documents were identified and searched. The words “infant”, “baby” and “babies” were identified in these plans and guidance 14 times.

Plans identified included the Commonwealth Disaster Response Plan (COMDISPLAN), Department of Health National Emergency Plans, and the Australian Emergency Management Arrangements. The COMDISPLAN provides a list of emergency expertise capability that the Federal government can provide to States and Territories. However, it does not identify any Commonwealth agency as having the capacity to provide expertise on IYCF-E [33]. The Department of Health Australian Health Management Plan for Pandemic Influenza repeatedly identifies infants as a vulnerable group in an influenza pandemic but contains no mention of breastfeeding as a measure promoting resilience and does not consider infant feeding management in the context of a pandemic [34]. The need to ensure safe food and water for emergency-affected populations is recognised the Australian Emergency Management Arrangements but the specific needs of infants and young children are not considered [35].

In regards to Federal emergency guidance, the 12 current handbooks of the Australian Institute for Disaster Resilience Collection were searched. The Community Recovery Handbook describes young children as a vulnerable group stating that, *“children and youth are uniquely vulnerable following an emergency event and require targeted and specialised support”* but notes that *“there is a lack of advocacy for children and youth in the emergency management arena”* [36]. The Community Recovery Handbook also emphasises that the provision of safe food and water to emergency affected individuals is a priority however, it does not identify the need for targeted feeding support for infants and does not provide guidance on how to ensure provision of food and water to infants and young children [36].

The Evacuation Planning Handbook states that children are vulnerable in evacuation centres but the existence of special needs in relation to food and water is not noted [35]. The National Strategy for Disaster Resilience *“focuses on priority areas to build disaster resilient communities across Australia”* however, it does not identify any health-related actions, such as breastfeeding, that build resilience [5].

The Disaster Health Handbook identifies children as vulnerable in emergencies and specifically notes their physiology, immune system and stage of development as predisposing factors [37]. It describes water and food as essential requirements after disasters. However, the water requirements described in the Handbook make no allowance for the needs of formula fed infants. Nonetheless, it notes that a shortage of infant supplies, including infant formula, have been experienced in past emergencies and that infant formula should be considered as a basic requirement in emergency shelters.

The National Guidelines for Managing Donated Goods notes that baby food and nappies are items that emergency-affected people say they require urgently following an emergency. It also notes that donations of goods should be discouraged because of logistical and waste issues but does not recognise that infant formula and other infant feeding products are a category of donation that can cause direct harm [38].

The Communities Responding to Disasters: Planning for Spontaneous Volunteers Handbook states that emergency organisations should establish relationships with special interest groups and work with them to identify roles and activities for volunteers but does not identify infant feeding related organisations as a potential source of volunteers [39].

### State and territory plans and guidance

A total of 192 State and Territory plans and guidance documents were searched. The words “infant”, “baby” and “babies” were identified 110 times. Content related to the vulnerability of infants and young children, importance of having plans for children, importance of food and water in emergencies, and infant and young child feeding were identified.

#### Vulnerability of infants and young children

Awareness regarding the vulnerability of infants and young children in emergencies is evident in some State and Territory plans. Plans noting that infants are a vulnerable group include those concerning pandemic, heat wave, chemical biological radiological, tsunami, health, flood, and black systems event emergencies as well as disaster recovery, evacuation, evacuation centre, social recovery, state emergency management, CBD emergency management, and welfare plans in a variety of jurisdictions. Texts concerning the vulnerability of infants and young children are generally extremely brief with little to no detail provided as to why and how they are vulnerable.

The vulnerability of infants and young children is also mentioned in several guidance documents including the Victorian Emergency Management Handbook and Psychosocial Support Framework as well as the South Australian People with Vulnerabilities in Disasters document [40–42]. Only one document, the Victorian Emergency Management Planning for Children and Young People focussed solely on the needs of children, providing a detailed explanation of why and how infants and young children are vulnerable in an emergency. It states, *“Children have distinct vulnerabilities in emergency and disaster situations, including unique physiological, psychological and developmental… Children rely on the care of adults; the level of care they need will depend on their stage in life. A newborn depends entirely on adults for its very survival… When an emergency occurs, children and young people may become more vulnerable if the adults who support them have also been affected by the emergency”* [43].

#### Importance of having plans and interventions for children

While many State and Territory documents note the vulnerability of children in emergencies, few state that emergency plans should be developed for children or that their needs should be taken into account in the development of emergency plans. Notable exceptions are the Western Australia State Emergency Management Plan and the Victorian Emergency Management Planning for Children and Young People guidance. The Western Australia State Emergency Management Plan states that, *“Children and youth may require special protection, both physical and psychological, in emergencies. The development of plans for children and youth should consider factors including: clear allocation of responsibility for the needs of children to specific roles or agencies; plans to maintain provision of essential services to children by agencies, organisations, educational and other facilities, especially those responsible for care and supervision of children; consultation with child protection experts by all levels of government”* [44]*.* However, while Western Australian plans commonly allocated responsibility for children who met specific criteria (for example, unaccompanied children, children in schools) no plan allocated responsibility to any agency for children overall or for infants in particular.

The Western Australia Community Evacuation In Emergencies Guidelines state that *“The Department of Communities will coordinate the provision of welfare support for evacuated persons attending evacuation centres…This will include specific arrangements for unaccompanied children, nursing mothers and other at risk persons”* [45]. However, the Department of Communities regional welfare plan searched as a part of the Local government plans search did not specify any arrangements for breastfeeding women, suggesting that this had not occurred [46]. The Victorian Emergency Management Planning for Children and Young People states that *“Children and young people are uniquely vulnerable and require targeted and specialised interventions to help ensure the best opportunity to achieve a successful recovery”* [43].

#### Importance of food and water in emergencies

State and Territory plans and guidance repeatedly state that food and potable water supplies are commonly disrupted in emergencies, that food and water are basic human needs required by emergency affected populations, and that provision of food and water should be a part of emergency response. “Water” was mentioned more than 1000 times and “food” mentioned more than 800 times in State and Territory emergency plans and guidance. Plans and guidance often note that food and water is provided in evacuation centres and nominate specific agencies as responsible for catering services. Provision of food and water to isolated individuals and communities as a part of emergency response was also commonly noted.

#### Plans and guidance related to infant and young child feeding

The specific food and fluid requirements of infants and young children and mechanisms by which their nutritional needs would be met were infrequently addressed in State and Territory plans and guidance. Content related to infant and young child feeding was most commonly located within documents dealing with evacuation centre/shelter management and resupply however, only Victorian and Queensland plans and guidance had such content. Queensland plans and guidance referred to the need for evacuation centre facilities for heating and refrigerating infant formula, a clean space for preparing baby food, and a need for resources for the “special needs of infants.” Infant formula is described as a “baby necessity” that evacuees should bring to evacuation centres and it is stated that pregnant and breastfeeding women will need additional water. It is noted that caregivers of infants should be prioritised in registration processes, that a private area should be provided for breastfeeding women who are described as a group with special needs. No agency is allocated responsibility for IYCF-E and some resources necessary for formula feeding (such as washing facilities and access to boiled water) are not mentioned. Breastfeeding support, assessment of needs and health support are not mentioned. Infant formula and baby food is noted as an essential item for resupply of isolated communities [47–50].

The Victorian Emergency Management Planning for Children and Young People states that appropriate food is needed for babies. It alerts planners of the need to consider the support needs of breastfeeding women, the supplies needed for formula fed infants and the need to ensure access to appropriate complementary foods for infants and young children. However, it does not specify what this support should consist of or what supplies are needed [43]. The Victorian Emergency Relief Handbook contains a section on evacuation centres stating that nutrition support requirements for infants and mothers should be considered during food and water planning, and that the ability to have a private space for infant feeding should be considered. A relief centre kit that includes baby bottles, baby food/formula and dummies (but not other necessary infant feeding resources) is also in this document. It is suggested that Local government areas should engage with community health organisations such as the local “Nursing Mothers Group” as a part of emergency health planning [51]. However, contact with the Victorian Branch of the Australian Breastfeeding Association (which was called the Nursing Mothers’ Association until 2001) revealed that no Local government area has so engaged.

Planning and guidance documents related to heat waves were notable because every State and Territory had documents stating that infants and children were vulnerable in heat waves. However, detail on how the specific needs of infants and young children should be accounted for was not provided in any State or Territory plan. Rather, general information not targeted at any particular group and which could result in harm to infants such as to *“drink plenty of water”* was contained within some plans [52]. The Victorian Heat Health Plan provided specific advice to those caring for children but did not separate out infants as a group that had different needs stating that children should “drink water regularly” in hot weather [53]. Guidance documents targeted at the general public sometimes provided detailed accurate information on the needs of infants for example *“Breastfed and bottle-fed babies less than six months of age will need to be fed more often in hot weather. Water or other drinks are not needed unless recommended by a doctor. Babies over six months of age can be offered small amounts of cooled boiled water, after or between milk feeds”* [54]. However, others provided only non-targeted information that would pose a danger to infants if followed, *“If there is a heatwave you should:… drink at least 2 to 3 litres of water per day, even if you’re not thirsty”* [55].

No State or Territory plan designated responsibility for IYCF-E with any agency or organisation.

### Local government area plans and guidance

Emergency management plans and guidance documents of eighteen Local government areas were searched. They were: Northern Grampians Shire, East Gippsland Shire, Latrobe City Council, Sorrell Council, Warrambungle Shire Council, Bundaburg Regional Council, Indigo Shire Council, Blue Mountains City Council, Bellingen Shire Council, Gunnedah Shire Council, Brisbane City Council, Baw Baw Shire Council, Buloke Shire Council, Cassowary Coast Regional Council, Melbourne City Council, Townsville City Council, Halls Creek, and Shire of Mundaring.

Local government area plans and guidance echoed State and Territory plans and guidance in commonly recognising the vulnerability of children in emergencies, and universally recognising the critical need for emergency affected populations to have access to safe food and water. They apportion responsibility for providing food to specific organisations such as the Red Cross, the Salvation Army and the Country Women’s Association. However, plans contained scant detail on the needs of children and no Local government area nominated an organisation as being responsible for IYCF-E or contained detailed planning or guidance in relation to IYCF-E.

### Emergency kits

Every Australian State and Territory provided advice to members of the public on recommended items for their household emergency kit. In most jurisdictions, information regarding the needs of infants was absent or lacked sufficient detail. The NSW, Victorian and ACT emergency kit lists contained no information directed to the needs of infants or young children [56–58]. In Tasmania, parents and caregivers were advised to include items for the *“special needs for babies”* [59, 60]. The South Australian emergency kit list noted that individuals who are caring for young children should consider their needs in compiling their emergency kit but the list of items includes only food, infant formula, drink, a change of clothes and nappies [61]. In the case of the Northern Territory, individuals are advised to pack *“for the special needs for infants (food, formula, nappies, toys)”* and also a portable cooker, gas, cooking equipment and eating utensils [62]. The Western Australian government emergency kit lists advise individuals to pack special items for infants, food, infant formula, drink, change of clothes, nappies, favourite toys, also cooking gear/eating utensils, portable gas stove/BBQ, and a container for boiling water [63, 64]. Queensland is the only jurisdiction to provide detailed information on emergency items for infants providing separate kit lists for breastfed infants and formula fed infants [65]. The emergency kit list for formula fed infants is extensive and includes detailed information on how to use the items in the event of an emergency. However, it only includes a 2 L of water per day allowance, which is insufficient. Most jurisdictions suggest that individuals pack water in their emergency kit but either do not specify an amount or note 3 L of water per person per day as the recommended quantity.

A Table summarising the presence and absence of important aspects of IYCF-E planning and guidance and the number of plans and guidance in each jurisdiction is presented in Table 1.

**Table 1:** Presence and absence of important aspects of IYCF-E planning and guidance and the number of plans and guidance in each jurisdiction

	National	New South Wales	Victoria	Queensland	South Australia	Western Australia	Tasmania	Australian Capital Territory	Northern Territory
Infants and young children recognised as vulnerable	X	X	X	X	X	X	X	X	X
Need for IYCF-E planning recognised			X						
Responsible agency/agencies for IYCF-E nominated									
Assessment of needs regarding IYCF-E provided for									
Breastfeeding counselling support provided for									
Support for caregivers of formula fed infants provided for									
Aspects of IYCF-E planning for evacuation centres included			X	X					
Management of donations of infant foods described									
Heat wave guidance distinguishes between infants under 6 months and older children or adults		X			X		X		
Heat wave guidance does not distinguish between infants under 6 months and older children or adults		X	X	X	X		X		X
Emergency kit list with detailed information on the needs of infants				X					
Number of plans and guidance	22	31	27	29	34	29	15	15	12

### Plans and guidance concerning animals

National, State/Territory, and Local government area plans and guidance contained extensive content related to the needs of animals. The words “animal” and “pet” were used 115 times in national plans and guidance and in excess of 2200 times in State and Territory plans and guidance.

Federally, the COMDISPLAN states that the Australian government provides expertise on animal welfare in emergencies to the States and Territories through the Department of Agriculture [33]. National guidance on animals in emergencies exists in the National Planning Principles for Animals in Disasters (NPPAD). This guidance contains recommendations that animals should be included in emergency preparedness, response, recovery and mitigation plans and that such plans should be developed with the involvement of animal experts. It says that these plans should designate the agencies responsible for animals and their roles, be included in emergency training, and be communicated to all stakeholders including the public. It also states that emergency arrangements with animal welfare organisations should be formalised. The National Planning Principles for Animals in Disasters notes that the guidance was developed by the National Advisory Committee for Animals in Emergencies (NACAE). It states that the NACAE has the goal *“to work collaboratively to incorporate animals into emergency management planning at all levels of government, and to encourage those responsible for animals in emergencies to accept their responsibilities*.” It notes that the Department of Agriculture facilitated the meeting that led to the formation of the NACAE.

The needs of animals were often considered in both the over arching State or Territory emergency management plan and in sub-plans dealing with specific emergency types or sectors. All States and Territories had plans that clearly designated the agency or agencies responsible for the welfare of animals in emergencies. All State and territories had plans regarding the evacuation or rescue of animals and content related to animals in evacuation centres. NSW, SA, Queensland and Victorian plans described the need for assessments regarding animal needs and NSW, Tasmania, Victoria and Queensland had plans for the provision of food for animals. Other areas of emergency response commonly mentioned in plans includes: arrangements where animals aren’t permitted in evacuation centres, management of assistance animals, lost animals, managing donations, managing volunteers, veterinary treatment, and foster care/agistment of animals.

In addition to these mentions in broader emergency plans and guidance, NSW, South Australia and Victoria have stand alone, extensive plans or guidance regarding animals in emergencies targeted at emergency responders. The content of these plans and guidance reflect the recommendations made in the National Planning Principles for Animals in Disasters [66–68]. The Northern Territory, Western Australia, Queensland and Tasmania had guidance only for the general public on animal welfare (however, Western Australia currently has a draft stand alone animal welfare plan under consultation). The ACT was the only jurisdiction that did not have plans or guidance for emergency planners/responders or the public regarding animals. It was common for Local government areas to have stand-alone detailed animal welfare plans with 10 of the 18 Local government areas included in searches having sub-plans specific to the welfare or management of animals in emergencies.

State and Territory and Local government area plans and guidance provide often very detailed information on how to ensure the welfare of animals in emergencies including for example, information on how to evacuate animals as diverse as dogs, cats, guinea pigs, mice, horses, lizards, snakes, fish, frogs, birds, and rabbits was identified.

The information on emergency items for pets in emergency kits varies widely between jurisdictions. Tasmania and the ACT do not mention pets in their emergency kit lists. NSW and the Northern Territory provide minimal information such as *“Food and medications for your pets”* [56]. Victoria, Queensland, Western Australia and South Australia either provide, or refer individuals to, detailed emergency kit lists specifically for pets including, in the case of South Australia, in the form of a video presentation [69].

## Discussion

Australian emergency plans and guidance contained numerous pointers to the desirability of having plans that address IYCF-E. Plans and guidance identified children as a vulnerable group and in need of targeted, specialist support [34–37, 40–43], stated that there should be plans for children [43, 45], stated that there should be arrangements made for breastfeeding women, that relationships should be developed with breastfeeding support organisations [45, 51], identified that inadequate support for IYCF-E has occurred in previous emergencies [37], and described the importance of providing safe food and water in an emergency [35].

However, the audit revealed a dearth of planning at all levels of government for the needs of infants and young children and for IYCF-E specifically. Where plans contained content related to infant feeding, they lacked sufficient detail, lacked important elements, or are demonstrated not to have been followed. This is not a new problem. A 2013 Save the Children Australia audit of emergency planning concluded that children suffer from “benign neglect” in emergency planning with their needs not routinely and systematically considered [70]. The question must be asked, why does Australia not have detailed IYCF-E planning especially given the high incidence of emergencies, the large numbers of infants with increased vulnerability due to formula feeding, and the existence of international and national direction that such plans should be developed [28, 29, 71]. The answer may be related to the fact that there is no agency at Federal or State/Territory levels of government with designated responsibility for children or for IYCF-E. As a result, it appears that no organisation has taken up the role of identifying and advocating for infants and young children or for ensuring that their needs are met, including in relation to food and water. This is not a problem of which the emergency management community is unaware, as was identified in this audit, the 2018 Community Recovery Handbook specifically described a lack of advocacy for children in emergency management [36].

The lack of agency responsibility and planning for children in general, and infants in particular, is in stark contrast to the situation regarding animals. Throughout Australia, and at all levels of government, emergency planning for animals is extensive with clear designation of organisational responsibility and with widespread comprehensive planning and guidance. Such guidance is sometime incredibly detailed, for example, providing a description of the sort of container that should be used to evacuate frogs. It seems not coincidental that plans and guidance commonly reflect the recommendations of the National Planning Principles for Animals in Disasters. The role of the NACAE in working *“collaboratively to incorporate animals into emergency management planning at all levels of government, and to encourage those responsible for animals in emergencies to accept their responsibilities”* has clearly been important. It is also significant that government departments with the designated responsibility for animals in emergencies have taken a lead role in the development and dispersal of these plans [66, 72]. Thus, the advocacy of the NACAE and government departmental responsibility and championing of animal welfare in emergencies, appears to have been central to the successful integration of animals in emergency planning.

However, there are other facts that may contribute to poor planning for infants and young children. The male dominance of the emergency management sector may be a factor in a lack of understanding of the support that mothers and caregivers need in relation to their infants and young children [73, 74]. Further, Save the Children proposed that planning for children may be lacking because planners assume that parents and caregivers will meet the needs of children and did not consider that parents and caregivers will need assistance in doing so [70]. What is concerning is that not only are government plans for IYCF-E inadequate, meaning that parents and caregivers of infants might not receive the support they require from agencies responding to emergencies, but emergency kits from all jurisdictions except for Queensland, contain no detailed information on the needs of infants in emergencies. This means that parents are not being supported to be prepared themselves. While breastfed infants generally need little in the way of supplies for feeding, formula fed infants require substantial resources in situations where there is no access to mains water or power, including a large amount of water [23]. Emergency kits for formula fed infants should list all of the items required in detail. Mothers of breastfed infants should be made aware of the assistance available to them via the National Breastfeeding Helpline.

Concerningly, the lack of consideration of infants has also led to heat wave guidance in many jurisdictions being dangerous to young infants. Because of their immature kidneys, feeding water alone to young infants in any significant quantity can cause hyponatraemia (water intoxication), which can result in brain swelling, seizures, brain damage and even death [75]. Infants who have gastroenteritis or are dehydrated (conditions occurring at increased prevalence in emergencies) are at increased risk of hyponatraemia [76]. However, heat wave emergency guidance commonly advises the consumption of water (sometimes in large quantities) without identifying that this guidance should not apply to young infants. Heat wave guidance should be urgently amended to differentiate the needs of infants, for example in line with that provided in NSW [54].

IYCF-E is a complex area of aid. Infants can be breastfed direct at the breast, fed expressed breastmilk, formula fed, or mixed breastfed and formula fed; all of these methods are common in Australia [77]. Mothers and caregivers need different assistance and resources to feed their babies depending upon how they are fed. Their needs also vary according to the age of their child. Lack of planning leaves infants and young children vulnerable. For example, the authors are aware of situations (including as relayed by an evacuation centre manager who provided assistance in this audit) where formula fed infants have arrived at evacuation centres where there was no infant formula. In these cases, breastfeeding women, who happened to be present, fed the babies. While this outcome was protective of the infants involved, emergency response should not rely on serendipity, to do so is simply irresponsible.

In relation to evacuation centres, it appears that catering services take the most prominent role in IYCF-E though the provision of infant formula. However, it might be asked if they are also responsible for ensuring access to boiled water for reconstituting infant formula, a clean preparation area, and access to washing facilities and resources (not in a bathroom) because these are also required to formula feed? And what happens when a breastfeeding mother reports difficulties with breastfeeding, who is responsible for helping her? No plans provided any detail on such matters. At an individual level, assessment of what feeding support is needed by caregivers requires an in-depth knowledge of infant feeding, including the physiology of lactation and the proper use of infant formula. This is a specialist role that can only be properly undertaken by a health worker with appropriate training. While such individuals may be found in existing health services and in the Australian Breastfeeding Association, current plans do not recognise the need for such support or provide a pathway for assistance. In the absence of IYCF-E planning, consistent support for breastfed and formula fed infants in evacuation and welfare centres is undoubtedly lacking.

For infants, lack of access to appropriate food and liquid for a relatively short period of time, particularly in hot weather, can result in dehydration, which can be fatal [78, 79]. Inappropriate feeding can result in other serious medical conditions (such a hyper or hypo-natraemia) [75, 80]. Infants exposed to contaminated water can develop life-threatening infections, especially if access to health care is impeded [81]. Infants unnecessarily weaned from breastfeeding, experience an acute increased risk of infection and longer term health and development impacts [82]. Lack of planning and guidance for IYCF-E is a public health issue that requires urgent attention in Australia. Given the complexities of IYCF-E and the depth of knowledge of infant feeding required, health agencies should take a lead role and responsibility for IYCF-E.

Action needs to be taken to ensure that emergency plans for IYCF-E are developed and integrated in Australia. The success experienced in the integration of planning for animals in emergencies is instructive. The following recommendations provide a potential model pathway to facilitating appropriate planning for IYCF-E in Australia:

- The Federal Department of Health should be designated in the COMDISPLAN as the resource agency providing advice and expertise to the States/Territories on IYCF-E. State and Territory governments should similarly allocate responsibility for IYCF-E to Ministries of Health.
- The Federal Department of Health should convene and appropriately fund a national advisory committee on IYCF-E with the purpose of incorporating the needs of mothers/caregivers, infants and young children into emergency management planning at all levels of government, and to ensure that appropriate agencies take responsibility for IYCF-E. This committee must include a broad range of stakeholders from all levels of government, health organisations and emergency management organisations.
- The Australian Institute for Disaster Resilience should produce a Disaster Resilience Handbook on IYCF-E and integrate cross-cutting IYCF-E issues into other Handbooks (e.g. Disaster Health, Planning for Spontaneous Volunteers, Evacuation Planning).
- Existing Australian education and training on IYCF-E (for example through the Australian Breastfeeding Association and the National Critical Care and Trauma Centre) to be made available to all relevant health and emergency workers.

Although this audit was of Australian emergency plans and guidance, it is apparent that IYCF-E planning in many other countries has been similarly neglected. A survey of 84 countries conducted by the World Breastfeeding Trends Initiative identified emergency preparedness was the least implemented aspect of infant feeding policy [83]. In addition, the World Health Organization Global Nutrition Policy Review identified that only 25% of 172 countries had an IYCF-E policy [84]. Undoubtedly, more attention to the needs of infants and young children in emergencies is required worldwide.

## Conclusions

Emergency planners in Australia have paid insufficient attention to the needs of infants and young children in emergencies placing them at risk of serious adverse health consequences in emergencies. Australian Federal, State/Territory and Local governments need to take action to ensure that IYCF-E plans and guidance are developed and deployed in line with international standards. The success of the development and integration of the needs of animals in emergencies provides a potential model pathway for a similar integration of IYCF-E plans. Government health authorities are best placed to take responsibility for IYCF-E but consultation and involvement of other sectors and community groups, such as the Australian Breastfeeding Association, should also be involved. Internationally, national governments should take action to ensure that their youngest, most vulnerable citizens are protected in emergencies through the development of appropriate IYCF-E plans.

## Data Availability

The dataset generated and analysed for this study is not publicly available as access to some of the emergency management plans included in the study is restricted by government policy and access to the authors was provided on condition of non-publication.

## Abbreviations

COMDISPLAN: Commonwealth Disaster Response Plan
IYCF-E: Infant and young child feeding in emergencies
NACAE: National Advisory Committee for Animals in Emergencies
OG-IFE: Operational Guidance on Infant and Young Child Feeding in Emergencies
